# PPARs and Metabolic Disorders Associated with Challenged Adipose Tissue Plasticity

**DOI:** 10.3390/ijms19072124

**Published:** 2018-07-21

**Authors:** Patricia Corrales, Antonio Vidal-Puig, Gema Medina-Gómez

**Affiliations:** 1 Área de Bioquímica y Biología Molecular, Departamento de Ciencias Básicas de la Salud, Facultad de Ciencias de la Salud, Universidad Rey Juan Carlos, Avda. de Atenas s/n. Alcorcón, 28922 Madrid, Spain; 2 Metabolic Research Laboratories, Wellcome Trust MRC Institute of Metabolic Science, Addenbrooke’s Hospital, University of Cambridge, Cambridge CB2 0QQ, UK; ajv22@medschl.cam.ac.uk; 3 Wellcome Trust Sanger Institute, Hinxton, Cambridgeshire CB10 1SA, UK

**Keywords:** PPAR, metabolism, adipose tissue, obesity, pregnancy, aging, caloric restriction

## Abstract

Peroxisome proliferator-activated receptors (PPARs) are members of a family of nuclear hormone receptors that exert their transcriptional control on genes harboring PPAR-responsive regulatory elements (PPRE) in partnership with retinoid X receptors (RXR). The activation of PPARs coordinated by specific coactivators/repressors regulate networks of genes controlling diverse homeostatic processes involving inflammation, adipogenesis, lipid metabolism, glucose homeostasis, and insulin resistance. Defects in PPARs have been linked to lipodystrophy, obesity, and insulin resistance as a result of the impairment of adipose tissue expandability and functionality. PPARs can act as lipid sensors, and when optimally activated, can rewire many of the metabolic pathways typically disrupted in obesity leading to an improvement of metabolic homeostasis. PPARs also contribute to the homeostasis of adipose tissue under challenging physiological circumstances, such as pregnancy and aging. Given their potential pathogenic role and their therapeutic potential, the benefits of PPARs activation should not only be considered relevant in the context of energy balance-associated pathologies and insulin resistance but also as potential relevant targets in the context of diabetic pregnancy and changes in body composition and metabolic stress associated with aging. Here, we review the rationale for the optimization of PPAR activation under these conditions.

## 1. Introduction

Approximately 39% of the world’s adult population is overweight and no less than 13% is obese. Obesity is currently the most prevalent chronic metabolic disorder and its current prevalence is predicted to triple by 2030 according to the World Health Organization (WHO). Beyond the obvious physical constraints and associated psychological stress, the main cause of morbimortality associated with obesity is its associated cardiometabolic metabolic pathologies, namely insulin resistance, dyslipidemia, and type 2 diabetes (T2D), a cluster of pathological entities globally designated as metabolic syndrome (MetS). Under normal circumstances, an excess of calories is considered advantageous for the organism as long as it is efficiently stored in the adipose tissue in the form of fat. However, excessive amounts of fat, beyond the available storing capacity of the adipose tissue (AT), or when accreted at a relatively fast pace, may overwhelm the functional capacity of the organ. When that happens, the excess of nutrients can, to a certain extent, be burnt, and/or alternatively be accumulated ectopically in other metabolically relevant organs, such as the liver, skeletal muscle, kidney, and pancreas—organs not purposely designed to be a main storage compartment. In these organs, as in the white AT (WAT), the excessive nutrient load induces metabolic stress causing lipid-related toxicity, a known cause for insulin resistance and inflammation [1,2].

Given these gloomy prospects, it has become increasingly necessary to identify pathogenic molecular mechanisms and diagnostic and prognostic biomarkers that can predict evolution and potential outcomes, as well as suitable therapeutic targets. Although metabolic syndrome has by definition different potential pathogenic entrances, we believe that given the relevance of its association with obesity, it is quite likely that in a high percentage of these predominantly obese patients, the dysfunction of their adipose tissue becomes a main contributor to subsequent associated complications. Peroxisome proliferator-activated receptors (PPARs) play important regulatory roles that control the homeostasis of the adipose tissue through the regulation of the balance between anabolic and oxidative processes. In this regard, we think that the PPARs associated with specific processes could be targeted, given their objective to beneficially improve insulin sensitivity, and that their agonists could be suitable candidates in the therapeutic arsenal to treat MetS.

PPARs are a group of ligand-activated nuclear hormone receptors. These transcription factors exist within a protein superfamily, which includes the receptors for retinoids, vitamin D, steroids, and thyroid hormones. These nuclear receptors bind to PPAR-responsive regulatory elements (PPRE) and heterodimerize with the retinoid X receptors (RXR), translocating to the nucleus where they contribute to transactivate and/or transrepress specific genes. In some respect, the PPARs are well placed to connect the environment represented by nutritional inputs [3,4] to specific genetic programs controlling genes involved in inflammation, adipogenesis, lipid metabolism, and glucose homeostasis [5].

There are three different isoforms of PPARs in mammals: PPARα, PPARβ/δ, and PPARγ. The three PPARs isoforms show structural similarities. However, despite their similarities, the isoforms exhibit differences in tissue distribution, ligand specificities, and functions. Recently, PPARs have been suggested to relate to the crossroads of obesity, diabetes, inflammation, and cancer [6]. Their topographic distribution and context-dependent regulation may be more important than the specific repertoire of genes they regulate, and collectively, they play an essential role in the maintenance of metabolic homeostasis [7,8,9].

PPARα is predominantly expressed in the liver and to a lesser extent in muscle, heart, bone, and brown adipose tissue (BAT), all of which are eminently prooxidative tissues rich in mitochondria content. In the liver, PPARα is activated under energy deprivation conditions. It is part of the adaptive response to fasting, and its main net contribution is to increase ATP production from β-oxidative phosphorylation, a process that requires coupling to the ancillary systems related to fatty acid transport and ketogenesis [10,11]. Moreover, the role of PPARα in controlling the expression of genes involved in lipid metabolism goes beyond its immediate effect of increasing energy availability in the liver, by also providing energy for supply to the peripheral tissues according to energetic demands in the heart, muscle, kidney, and brown AT during fasting. Through its prooxidative anti lipotoxic effects, PPARα ligands are successfully used therapeutically to treat primary and secondary forms of hypertriglyceridemia particularly associated with MetS [12,13].

PPARβ/δ is ubiquitously expressed but is particularly active in skeletal muscle, where it contributes to sustain the energy requirements for physical exercise by upregulating fatty acid β-oxidation specifically during fasting [14]. This PPAR isoform is also expressed in adipocytes and macrophages, where it reduces the expression of proinflammatory markers, such as nuclear factor kappa B (NF-κB), conferring the systemic anti-inflammatory activity of this isoform [15,16].

PPARγ is predominantly expressed in adipose tissues, both white and brown, where it plays an important anabolic role in facilitating fat storage, adipogenesis, and thermogenesis [17,18]. There are two main isoforms, PPARγ1 and PPARγ2, differentiated by an extra exon of 90 nucleotides in the end terminus of the γ2 isoform. PPARγ1 has a widespread distribution and seems to support a sort of housekeeping metabolic role, which is particularly relevant in the intestine, macrophages, the liver, muscle, pancreatic β-cells, bone, placenta, and adipose tissue. Conversely, the expression of the PPARγ2 isoform is restricted under physiological conditions to adipose tissues. However, under conditions such as long-term overnutrition or obesity, PPARγ2 is induced de novo in the liver and skeletal muscle, in parallel with the development of ectopic accumulation of lipids in these and other organs [19]. Both PPARγ isoforms contribute to the uptake of glucose and lipids, and when expressed ectopically, they promote safe deposition of lipids in peripheral tissues, such as the liver, muscle, and adipose tissue. When interpreting the role of PPARγ isoforms on the maintenance of energy homeostasis, it is important to consider the effect that the ectopic induction of PPARγ2 contributes to facilitating the reorganization of the inter-organ communication of nutrients and energy fluxes, which will help to understand how, when defective, it may lead to insulin resistance [20]. PPARγ has been heavily studied in part because the availability of its pharmacological agonists (TZDs) ligands, such as rosiglitazone and pioglitazone, both known to improve insulin resistance and exert anti-inflammatory effects in the adipose tissue [21,22,23] and on a systemic level. Such effects are potentially important in the treatment of obesity and T2D but also could have therapeutic value in physiological states, such as pregnancy and aging, characterized by insulin resistance and changes in body composition.

In this review, we summarize the contribution of PPARs to the maintenance of the adipose tissue physiology and discuss the pathogenic role mediated by dysfunctional PPARs in different contexts, characterized by defective adipose tissue expandability or functional failure associated with the development of insulin resistance and T2D, such as obesity, pregnancy, and aging.

## 2. Adipose Tissue Physiology and Lipotoxicity

Two main types of adipose tissue—white and brown adipose tissue (WAT and BAT, respectively)—exist. WAT is an endocrine organ that stores and mobilizes energy reserves as fat, whereas BAT uses lipids to produce heat by promoting uncoupled fatty acid oxidation converting nutrients in heat upon β-adrenergic stimulation or cold exposure. Both white and brown are necessary and contribute to maintain whole-body energy homeostasis [24].

Beyond its storage function, the WAT is an important endocrine organ responsible for synthesizing hormones, chemokines, and cytokines that modulate food intake, insulin sensitivity, or inflammation, which contribute to the maintenance of whole metabolism functionality [25,26]. In healthy conditions, the main function of the subcutaneous WAT is lipid storage of free fatty acids (FFAs) as triglycerides (TGs) in large unilocular droplets. However, in the context of chronic energy surplus leading to weight gain, the subcutaneous WAT adapts by increasing the cell size of the existing adipocytes (hypertrophy) and/or increasing the number through differentiation of new adipocytes (hyperplasia). Initially, this adaptation is sufficient to store lipids in the WAT, preventing them from ectopically accumulating in the liver or muscle. But, once adipose tissue storage capacity is exceeded above an individualized threshold, where the subcutaneous WAT cannot accommodate the excess of lipids, then these lipids are ectopically deposited in the liver, pancreas, muscle, kidney, and other important peripheral tissues. As obesity progresses the adipose tissue becomes inflamed and fibrotic, further contributing to the dysfunction of the AT. Both the failure to take upon lipids and to appropriately mobilize them decreases the metabolic flexibility of the WAT and exerts a knock-on effect on other organs leading to the development of metabolic abnormalities, such as dyslipidemia and peripheral insulin resistance [24,27].

In contrast to the unique, large lipid droplets of white adipocytes, BAT stores TGs in multilocular lipid droplets. This distribution of lipids in small vesicles helps to titrate the release of lipids destined to be oxidized by the mitochondria to produce heat through “uncoupling” of oxidative phosphorylation of FFAs stimulated by β-adrenergic sympathetic nervous system (SNS) typically observed in cold exposure. By oxidizing nutrients, BAT activation counteracts obesity, reduces TGs in the plasma, and reduces atherosclerosis development [28,29]. Furthermore, another type of thermogenic adipocytes can also be found interspersed in white fat depots, such as cells known as ‘brite/beige’ adipocytes [30]. This type of adipocytes appears to respond to thermogenic stimulation and, in principle, are expected to contribute to the regulation of body weight and the improvement of insulin resistance [31]; however, their functional relevance has not been clearly demonstrated so far.

Although there is a correlation between fat mass and insulin resistance, the adipose tissue mass by itself is not the main determinant factor linking obesity and insulin resistance. The relative mismatch between storage capacity and lipid load supply could be considered to be more relevant. By upregulating the capacity of adipose tissue to expand and store and/or by decreasing the supply of lipids, we may tweak this balance, preventing the mismatch. PPARs are key transcriptional regulators of this balance by contributing to increase the storage capacity of the adipose tissue and also, through their prooxidative effects, to decrease the demand for storage or the supply of lipids. A second layer of complexity comes from the knock-on positive effect, that by restoring the balance of the AT, it will exert an influence on the function of other metabolic tissues as a result of removing the toxic effects determined by ectopic fat deposition.

## 3. PPARs and Fat Mass Expansion and Function

**Figure 1:**
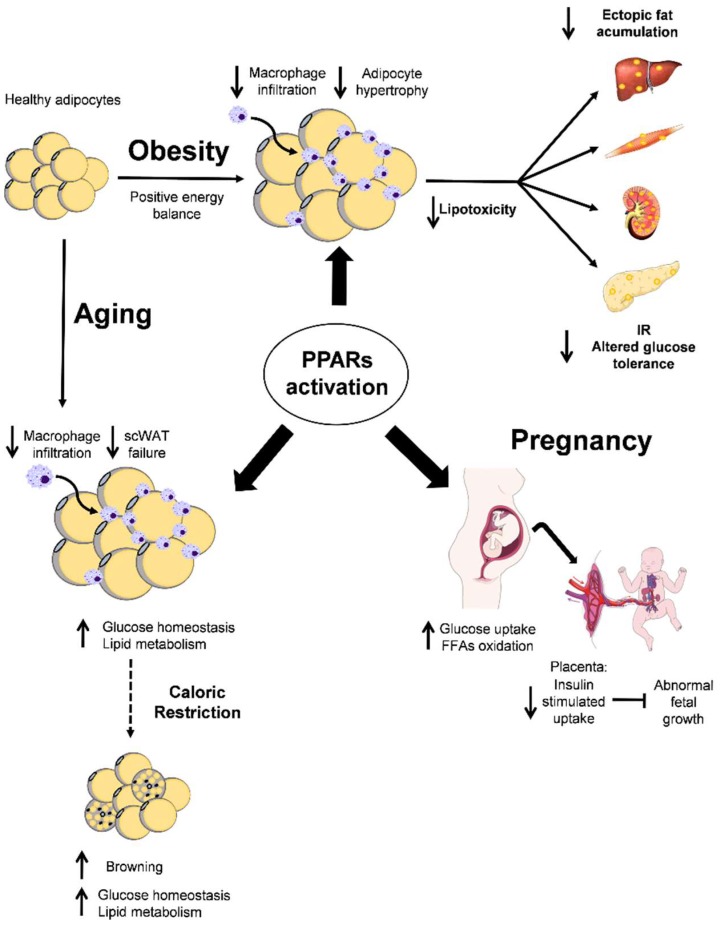
Overview of the effects of peroxisome proliferator-activated receptors (PPARs) activation in obesity, pregnancy, and aging. In obesity, PPARs activation decreases fibro-inflammation and ectopic fat accumulation in the adipose tissue (AT). In pregnancy, PPARs activation stimulates glucose uptake and fatty acid oxidation in the mother, while the placenta decreases insulin uptake to ameliorate abnormal fetal growth. In aging, this PPARs activation increases the glucose uptake and lipid metabolism in the subcutaneous white AT (scWAT). Moreover, both PPARs activation and caloric restriction (CR, dotted arrow) in aging promote browning in the AT, which improves the whole-body metabolism. The final effect of PPARs activation in all situations is the improvement of insulin resistance (IR). 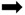 PPARs activation; 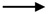. effects or PPARs activation; 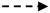 CR effect; 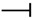 inhibition; 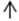 increase or 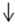 decrease effect.

The binding of ligands to PPARγ results in molecular changes, including the dissociation of co-repressors and the recruitment of co-activators, ultimately leading to changes in the coordinated expression of networks of genes functionally linked to adipogenesis, lipid metabolism, inflammation, thermogenesis, and body glucose homeostasis. PPARγ activation facilitates fat accretion and retains the functionality of adipose tissue by coordinating adipogenesis, fat transport, and lipolysis upon reaching an individualized threshold of adipose tissue mass. Defects in PPARγ, either in the form of mutants or secondary-to-decreased expression, compromises adipose tissue function, plasticity, and lipotoxicity. This is accompanied by the development of peripheral insulin resistance and ultimately global metabolic disruption. Restoration/maintenance of PPARγ functionality senses the lipid load and enables the recovery of the homeostasis of essential metabolic pathways (Figure 1).

PPARα is preferentially expressed in liver, where its activation is essential to generate energy, particularly under conditions of energy deprivation paradigms by promoting fatty acid uptake and oxidation [10]. This isoform exerts a pleiotropic effect controlling liver glucose metabolism, as observed in mice where PPARα activation by fibrates decreases expression levels of glucokinase [32] and suppresses pyruvate transformation to acetyl-CoA [33]. PPARα is also expressed in the WAT, where its activation elicits systemic effects in rodents by decreasing adiposity and ameliorating the insulin resistance in obese mouse models [34]. Moreover, it has been reported that treatment with PPAR-α agonists also increases the expression of adiponectin by the WAT [34,35] and decreases the tumor necrosis factor α (TNF-α) levels [36,37]. This anti-inflammatory effect in the WAT suggests PPARα activation has the capacity to improve insulin resistance and ameliorate obesity. In the BAT, PPARα exerts a thermogenic effect, cooperating with the peroxisome proliferator-activated receptor gamma coactivator 1-alpha (PGC1α) to control lipid oxidation and thermogenesis in response to β-adrenergic stimulation in response to cold exposure [38]. Moreover, activation of PPARα in obese mice increases energy expenditure and activates thermogenic pathways that facilitate weight loss [39]. Because of PPARα agonists’ prooxidative actions, activators of this nuclear hormone receptor may be used to improve obesity-induced insulin resistance.

PPAR-β/δ in the BAT regulates the fatty acid oxidation [14,40] and the thermogenic response contributing to the induction of the uncoupling protein 1 (Ucp1) expression and leading to the reduction of the WAT mass [40]. Moreover, this isoform may also have an anti-inflammatory effect when activated [41,42]. The metabolic function of PPAR-β/δ in the WAT has been much less studied, although it is known that this isoform facilitates preadipocyte differentiation [43]. Nowadays, PPAR-β/δ activators are under study for their clinical advantages in treating obesity.

PPARγ is expressed predominantly in the adipose tissues, where it acts as sensor of lipids, hormones, vitamins, and endogenous metabolites. This isoform is an important regulator of adipocyte differentiation, fat storage as triglyceride, and energy homeostasis. Both isoforms of PPARγ, PPARγ1 and PPARγ2, are necessary for the adipogenic function, and alteration in their expression increases susceptibility to lipodystrophy, insulin resistance, and T2D [44]. PPARγ is necessary for fat cell differentiation in all adipose depots and contributes to define the maximum threshold of expansion of the WAT. This is supported by studies showing that ectopic presence of PPARγ in non-adipogenic cells trans-differentiates them into mature adipocytes [45,46]. Moreover, PPARγ-deficient mice cannot develop adipose tissue [47,48]. Mice with PPARγ knockout in mature cells also develop insulin resistance and hyperlipidemia through dysregulation of molecular pathways of insulin signaling, FFAs uptake, and lipolysis [49]. Specific knockdown of the PPARγ2 isoform in mice results in adipose tissue dysfunction and insulin resistance [2]. Moreover, when the adipose tissue of this model is challenged with increased lipid supply, as characteristically observed in a leptin-deficient obese (*ob*/*ob*) background (POKO mouse [50]), these mice are precociously more insulin resistant, as young as 4 weeks, an age where the differences in fat mass in comparison with an *ob*/*ob* mice are not well established. In addition, the POKO mice became diabetic and hyperlipidaemic at 16 weeks of age, despite weighing less and having less fat than an *ob*/*ob* mouse at that age, with increased toxic reactive lipid species in different tissues, behaving like a mouse model of lipotoxicity and metabolic syndrome [51]. This reinforces the concept that it is not the absolute amount of fat mass, but the mismatch between nutrient supply and storage capacity that results in dysfunctional adipose tissue and metabolic stress. Furthermore, in states of obesity, the expression of PPARγ decreases with the consequent induction of a high grade of inflammation, angiogenesis, and fibrosis in the WAT [52] and low levels of adiponectin, which limits the adipose tissue expansion. Consistent with this, patients with mutations of PPARγ develop lipodystrophy and insulin resistance [53]. Conversely, increased expression of PPARγ protects from the insulin resistance associated with obesity [54].

TZDs, the pharmacological agonists of PPARγ, have been used clinically as antidiabetic agents, and their beneficial effects are well documented in relation with insulin resistance and obesity. The activation of PPARγ by TZDs in the WAT improves WAT expansion, alleviates peripheral lipotoxicity and normalizes adipokine secretion [24]. This activation improves the WAT’s ability to store lipids and reduces lipotoxicity in the liver and muscle by the activation of metabolic pathways implicated in FFA oxidation. The metabolic effects include lower levels of TGs in circulation, in the liver, and muscle, coupled with increased TGs in the adipose tissue [52]. The expression of TNF-α is also inhibited using TZDs [55]. Furthermore, PPARγ stimulates adiponectin production in the WAT, which contributes to further stimulating FFA oxidation, reducing hepatic glucose, and increasing the use of glucose by muscle [56]. Recently, it has been shown that PPARγ activated by TZDs promotes the expression of the fibroblast growth factor family (FGF1 and FGF21) showing the key role of the PPARγ–FGF axis, which contributes to the remodeling of the adipose tissue and the maintenance of metabolic homeostasis [57,58]. Thus, the result of pharmacological intervention in obesity with TZDs is the improvement of insulin sensitivity derived from the effects of TZDs improving adipose tissue function despite the associated increase in fat mass.

TZDs can promote browning in the WAT. Activating PPARγ [59] increases the expression of BAT-specific genes, such as *Ucp-1* and *Prdm16* [60], via *Sirt1* [61], priming the oxidative capacity of the adipose tissue through its transformation into brown-like adipocytes. These effects confer thermogenic properties by promoting mitochondrial biogenesis in the WAT, which can help in the remodeling of the adipose tissue and insulin resistance improvement under obesity conditions.

Therefore, the role of PPARγ improving glucose metabolism and insulin sensitivity is well established and provides insights into the molecular regulation of adipose tissue expansion in normal and obese/lipodystrophy pathological states but also in other situations in which the adipose tissue is physiologically stressed, such as pregnancy and aging.

## 4. PPARs and Pregnancy

Pregnancy involves hormonal and metabolic adaptations that directly affect maternal adiposity. In the early stages of pregnancy, the adipose tissue mass expands due to an increase in the lipid accumulation (known as a primarily anabolic phase). There is an increase in lipid synthesis and fat storage that prepare the mother’s metabolism for the prospective increase in fetal energy needs at a later phase. This increase in lipid/energy supply is enabled by maternal hyperphagia and improved insulin sensitivity, which stimulates FFAs synthesis in adipocytes and the uptake of FFAs from circulating TGs for preferential accumulation in the adipose tissue. Moreover, the production of hormones, such as progesterone, cortisol, and leptin, also contribute to facilitated fat storage and adipocyte hypertrophy. However, in contrast to this early anabolic phase of gestation, the adipose mass decreases in a later phase (known as a net catabolic phase). During the late phase of gestation, IR and a low grade of inflammation, especially in the adipose tissue, are developed, which should be considered as a physiological adaptation. Moreover, the decrease in insulin sensitivity enhances lipolysis, helping to mobilize the stored TGs. The human placental lactogen (Hpl) also stimulates lipolysis in adipocytes, coupled with the decrease in FFAs uptake from TGs in the plasma. The net result of these changes is a reduction in the adipose tissue mass and an increased glucose flux from mother to fetus. Although the physiological IR developed in the late phase of pregnancy is well documented, the mechanisms causing the changes in the adipose tissue and insulin resistance during pregnancy are still unclear.

Together with the exacerbated insulin resistance, insulin secretion may also become inadequate to meet the increased demands in the late stage of pregnancy, leading to gestational diabetes mellitus (GDM). Moreover, their offspring have an increased risk of perinatal complications, obesity, and diabetes in adulthood [62]. GDM is defined as glucose intolerance on first recognition during pregnancy [63] and characteristically shows altered plasma adipokine levels, inflammation, deregulation of the insulin signaling pathway, and oxidative stress [64,65,66]. The mechanisms underlying the GDM are not fully understood; however, it is known the association between inappropriate PPARγ function/levels and GDM through its function in both the adipose tissue and the placenta [67,68].

During early pregnancy, as in obesity, the mechanisms leading to energy storage and adipose tissue expansion are activated. It has also been reported that in advanced pregnancy, PPARγ declines, thus accelerating adipose tissue insulin resistance and facilitating lipolysis in the subcutaneous adipose tissue of obese pregnant women with GDM [67]. Moreover, PPARα and PPARβ/δ expression also decreases in adipose tissue from obese pregnant women and/or women with GDM [69]. In pregnant mouse models, the association between a decrease in PPARγ expression, exacerbated lipolysis in the AT [70], and the subcutaneous AT dysfunction has been reported. In agreement with this, we have shown that genetic ablation of PPARγ2 in pregnant mice is associated with poor AT expandability and the worsening of insulin resistance [71]. The contribution of PPARγ2 is also important for the process of pancreatic β-cell mass expansion and adaptation in murine models of MetS [50,71,72]. A missing study is the PPARγ deleted specifically in the pancreatic β-cell in order to study the mechanisms implicated in its adaptation when pregnancy occurs. Furthermore, it has been reported that the use of PPARγ agonists reverse the insulin resistance associated with late pregnancy in murine models [65]. For this reason, more studies are necessary to elucidate the potential of PPARγ agonism to overcome defects in pregnancy related to insulin resistance and GDM.

The role of PPARs in the placenta is potentially important. The placenta is an endocrine gland that synthesizes the peptides and steroid hormones during pregnancy that are essential for the maintenance of mother and fetus. Regarding their roles, PPARα null female mice become diabetic during pregnancy and have an increased risk of spontaneous abortion [73]. PPARδ also has a relevant role in embryonic, decidual, and placental function [74]. Moreover, PPARδ and PPARγ null mice are not viable and exhibit a failure in the development of the placenta [75]. PPARγ is downregulated in the placenta in human patients with GDM. PPARγ has anti-inflammatory effects in the placenta and modulates embryogenesis, implantation, trophoblast invasion, and maternal spiral artery transformation [76]. Moreover, it has been demonstrated that reduced expression of PPARγ in placental tissues and serum is contributing to the development of preeclampsia, a specific pregnancy disorder in humans that contributes to maternal mortality [77]. However, in a mice study, PPARγ expression was upregulated in the placentas of diabetic pregnant mice [78]. These contradictory effects may lead to specie-specific effects and would need to be further elucidated to be used to improve the GDM in pregnancy.

## 5. PPARs and Aging

Aging is a complex and multifactorial progressive physiological decay process, associated with an increased risk of metabolic disorders, such as obesity, insulin resistance, and other manifestations related to MetS, which are exacerbated by age. Moreover, aging is associated with changes in body composition characterized by increased total adiposity and topographical redistribution of adipose tissue defined by preferential loss of the subcutaneous WAT coupled with expansion of adipose in the visceral compartment [79,80]. Accretion of visceral, rather than subcutaneous WAT has been associated with the development of insulin resistance. The expansion of the intraabdominal adipose tissue may also be considered another example of peripheral lipotoxicity determined by a primary defect in the subcutaneous WAT. An important concept is the fact that as we age, the adipose tissue ages as other tissues, such as muscle or the liver, do. When muscle becomes frail/sarcopenic, with decreased oxidative capacity, lipids are redirected to the adipose tissue for storage precisely at a time when the adipose tissue itself has aged and is less functional and competent to deal with increasing metabolic challenges. In this regard the adipose tissue of the aged individual is subjected to even more lipid load, increasing the chances of AT dysfunction and peripheral lipotoxicity. In fact, ectopic fat accumulation in muscle is observed in lean elder individuals.

This conflict between increased storage demand and age-related decay results in increased adiposity coupled with macrophage infiltration and inflammation that interferes with insulin signaling. Thus, a key factor determining the shift of the AT towards the inflammatory state is the mismatch between the demand for storage and capacity [81]. Moreover, as inflammatory cells can be high-level producers of selective fibrotic molecules [82]**,** the old adipose tissue characteristically shows age-related fibro-inflammatory changes. Fibrosis is a disease process that deposits collagen-rich extracellular matrix (ECM) in an attempt to remodel and repair the tissue morphology and organ functionality of a failing organ. Transforming growth factor β (TGF-β) plays a key role in fibrosis, modulating the balance between the rate of synthesis and the degradation of matrix collagen proteins. It has been reported that a role for PPARγ is as a potent antifibrotic factor in the kidney [83]. In the AT, it has also been reported that TGF-β, apart from increasing collagen deposition, also increases mechanical stress on the adipocyte membrane and the rigidity of AT, compromising its further expansion. This rigid matrix can result in cell death by necrosis. In this situation, PPARγ agonists decrease collagen levels in the AT and confers a more flexible environment for the adipocyte growth and remodeling [84]. Thus, we could speculate that age-related defects in the adipose tissue remodeling may also contribute, particularly in the context of obesity, to exacerbated pathological conditions linked to insulin resistance [85].

Based on this evidence it is conceivable that age-related changes in PPARs may contribute to some of these pathological changes. However, at present, there is a paucity of information about how PPARs activation may contribute to delay or ameliorate these pathological changes.

Given the importance of PPARγ in adipose tissue biology, it is important to determine the contribution of PPARγ dysfunction in aging-associated metabolic decline. It is well documented, the role of PPARγ in coordinating gene expression programs of adipocyte differentiation, lipid storage, and lipolysis. Previous reports also suggest that PPARγ deficiency selectively in the subcutaneous AT during aging is associated with increased AT expansion that is associated with the development of insulin resistance [86]. Moreover, other studies have shown that oxidative stress and reactive oxygen species (ROS) production (characteristically observed in aging and linked to insulin resistance in adipocytes) modulate other proinflammatory pathways, linking PPARγ dysfunction with inflammation [87], such as NF-κB [88], likely to contribute to altered tissue expansion and inflammation and associated age-related insulin resistance. In addition, mitochondria, as the core organelle required to maintain cellular functionality and glucose and lipid homeostasis [89], have been suggested as key contributors to adipocyte formation through ROS signaling [90]. In aging, as in obesity, mitochondrial enzyme expression is reduced in the AT leading to decreased oxygen consumption and oxidative phosphorylation [91,92] in response to lipid overload, usually coupled with decreased AT insulin sensitivity [93]. Interventions with TZDs induce mitochondrial biogenesis, ROS, and remodeling in the AT, enhancing fatty acid oxidation and oxygen consumption, which seems to contribute to changes in the whole-body energy metabolism and insulin sensitivity [94]. Moreover, insulin resistance in elderly patient populations has been associated with decreases in mitochondrial oxidative phosphorylation [95]; however, further work is required to identify the mediators of nuclear-encoded mitochondrial genes induced by PPARγ ligand-dependent mechanisms that could be helpful during aging.

Furthermore, both PPARα and PPARγ decrease in the kidney with aging. This is correlated with accelerated oxidative stress and counteracted by the antioxidative action of caloric restriction [96]. All these studies suggest that defective PPARs are important for the defects in energy expenditure and lipogenic function leading to lipid accumulation in the whole body during aging. From this, it is conceivable that targeting these isoforms could be a helpful approach to reduce or prevent age-associated metabolic decline and protect from lipid accumulation and lipotoxicity. It could be speculated that TZDs treatment may help to reduce the side effects of weight gain in the elderly by minimal/function specific PPARγ activation stimulating insulin sensitivity without promoting adipogenesis [97]. But, in any case, it would be important to overcome some of the negative effects observed when using TZDs in the elderly. Amongst them is the increased bone marrow adiposity and reduced bone formation, resulting in osteopenia, bone fracture, and other complications [98]. Moreover, the use of some PPAR agonists had to be withdrawn from the United States (US) and European markets because of associated complications, such as edema, weight gain, macular oedema, heart failure, and bladder cancer, that have been associated side effects [99]. These are important drawbacks but potentially addressable with increased knowledge of the specific PPARα dependent pathways mediating them.

An added value of the PPARα agonists occurs in the muscle. Old age is associated with dyslipidemia, which together with the increase and dysfunction of the AT can lead to preferential deposition of TGs in skeletal muscle, ultimately leading to IR. Agonists of PPARα and PPARβ/δ have been used to treat dyslipidemia by increasing oxidative capacity in muscle fibers and improving insulin sensitivity [100]. Furthermore, TZDs as specific activators of PPARγ are used as insulin sensitizers and as regulators of FFAs storage, which may prevent intramuscular lipid accumulation [80] and maintain skeletal muscle insulin action [101].

Caloric restriction (CR) is another therapeutic paradigm representing a non-pharmacological intervention to efficiently delay the deleterious effects of age-related metabolic diseases [102]. Previous studies in animal models have shown that CR exerts physiological effects leading to reduced body weight and glucose and insulin serum levels [103]. Moreover, the reduction of adiposity by CR [104] or fat removal [105] have been demonstrated to ameliorate age-associated insulin resistance. Of note, CR alters the expression of genes that are regulated by PPARs and that are involved in lipid metabolism and insulin signaling. In some ways, the beneficial effects derived from fasting may be mediated, at least in part, by these nuclear receptors [106]. The effects of CR in the AT on PPARα and PPAR-β/δ have not been shown yet, but it is known that CR and PPARγ agonists can improve the reduced mitochondrial function in the WAT due to aging and obesity [107]. Moreover, it has been reported that CR induces BAT functionality [102]**,** and it has been speculated that the induction of a brown fat phenotype in the WAT by CR or PPARγ agonists would result in an increased mitochondrial functionality with beneficial effects on aging and metabolism. Due to the improvement in aging conditions by using PPARs agonists, more studies are needed to document the role of PPARs on adipose tissue plasticity during aging.

## 6. Conclusions

Defective adipose tissue synergizes with the age-related metabolic defects to exacerbate metabolic diseases. Thus, the understanding of cellular mechanisms governing the plasticity of adipose tissue should help to understand and provide therapeutic rationale to address metabolic disorders. Understanding the molecular alterations that determine the impaired adipose tissue plasticity may identify therapeutic targets to optimize AT expandability and function. Thus, PPARs should be considered candidates to improve age-related metabolism through their influence in the balance between anabolic and catabolic processes and by limiting unwanted inflammatory reactions that may compromise lipid and glucose homeostasis. Thus, PPARs may have the clue to restore, delay, and improve the metabolic balance in those conditions that render someone particularly susceptible to developing insulin resistance, such as obesity, pregnancy and aging.

## Abbreviations

T2D	Type 2 diabetes
MetS	Metabolic Syndrome
AT	Adipose tissue
PPARs	Peroxisome proliferator-activated receptors
PPRE	Ppar response element
RXR	Retinoid X receptor
NF-κB	Nuclear factor kappa B
TZDs	Thiazolidenidiones
WAT	White adipose tissue
BAT	Brown adipose tissue
FFAs	Free fatty acids
TGs	Triglycerides
TNF-α	Tumor necrosis factor α
PGC1α	Peroxisome proliferator-activated receptor gamma coactivator 1-alpha
Ucp-1	Uncoupling protein 1
GDM	Gestational diabetes mellitus
CR	Caloric restriction
